# Risk and protective factors of acute kidney injury in decompensated cirrhotic patients with ascites on tolvaptan

**DOI:** 10.1002/jgh3.12672

**Published:** 2021-11-01

**Authors:** Tomomi Kogiso, Yuri Ogasawara, Takaomi Sagawa, Makiko Taniai, Katsutoshi Tokushige

**Affiliations:** ^1^ Institute of Gastroenterology, Department of Internal Medicine Tokyo Women's Medical University Shinjuku‐ku Tokyo Japan

**Keywords:** acute kidney injury, kanamycin/rifaximin, liver cirrhosis, proton pump inhibitor/H2 blockers, tolvaptan

## Abstract

**Background and Aim:**

Acute kidney injury (AKI) is a life‐threatening complication of liver cirrhosis. Here, we evaluated the risk factors and characteristics of AKI in cirrhosis.

**Patients/Methods:**

This was a single‐center retrospective study. A total of 199 Japanese patients with decompensated liver cirrhosis (104 men, median age 61 years) were enrolled and received tolvaptan orally. Survival rates and new onset of AKI were monitored, and risk factors were evaluated.

**Results:**

Forty‐six patients (23.1%) suffered an AKI complication and exhibited significantly poorer survival (*P* < 0.01). The rates of hepatic encephalopathy (*P* < 0.01) and chronic kidney disease (CKD; *P* = 0.02) were significantly increased in patients with AKI. The rate of proton pump inhibitor (PPI)/H2 blocker treatment (*P* = 0.04) was significantly lower, whereas that of ascites drainage was significantly higher in the AKI cases (*P* < 0.01). The AKI risk was significantly increased in patients with hepatic encephalopathy (HR 4.18, 95% CI 1.618–10.771). In contrast, the incidence of AKI was significantly lower in patients with a higher serum albumin level (HR 0.36, 95% CI 0.142–0.914, *P* = 0.03). Treatment with PPI/H2 blockers (HR 0.30, 95% CI 0.126–0.711, *P* < 0.01) or kanamycin/rifaximin (HR 0.26, 95% CI 0.075–0.929, *P* = 0.04) was significantly associated with a reduced risk of AKI development.

**Conclusions:**

AKI incidence was increased in patients with decreased liver function and was associated with poor survival. PPI/H2 blocker or kanamycin/rifaximin treatment may reduce the risk of AKI.

## Graphical abstract

In cirrhosis, AKI incidence was increased in patients with encephalopathy and poor liver function. While proton pump inhibitor/H2 blocker or kanamycin/rifaximin treatment may reduce the risk of AKI.

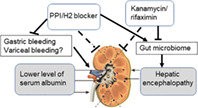

## Introduction

Acute kidney injury (AKI) is a common and frequently life‐threatening complication of liver cirrhosis.^1^ The incidence of AKI has been reported to be as high as 20–50% among patients with liver cirrhosis.^2^, ^3^ In addition to prerenal azotemia, AKI patients have been reported to develop hepatorenal syndrome (HRS) and acute tubular necrosis.^2^ In 2012, the Kidney Disease: Improving Global Outcomes (KDIGO) group proposed new definitions of AKI based on creatinine elevation or the disturbance in urination.^4^ The International Club of Ascites (ICA) then adapted these definitions for patients with cirrhosis (ICA‐AKI criteria) in 2015.^5^

When liver disease progresses, decreased renal perfusion causes activation of the renin–angiotensin–aldosterone system, resulting in sodium and water retention and extrasplanchnic vasoconstriction.^2^ Liver cirrhosis is associated with the induction of vasodilators such as nitric oxide, carbon monoxide, and endogenous cannabinoids as well as vasodilation due to inflammatory cytokines such as tumor necrosis factor‐α and interleukin‐6 induced by and translocated from bacterial peritonitis in the gut.^6^ High portal pressure and severe vasodilatation predispose patients with cirrhosis to increased risk of AKI and chronic kidney disease (CKD).^7^ The use of AKI‐precipitating medications was reported to be the most common cause of AKI, followed by bacterial infection, including spontaneous bacterial peritonitis (SBP).^8^

The recommended treatment for cirrhosis has been updated in the Clinical Practice Guidelines for Decompensated Cirrhosis proposed by the European Association for the Study of the Liver (EASL) in 2018.^9^ The Japanese Society of Gastroenterology and the Japan Society of Hepatology also revised the guidelines for the treatment of cirrhosis in 2020.^10^ Overdosing with diuretics should be avoided, and use of the vasopressin V2‐receptor antagonist, tolvaptan, was recommended prior to renal impairment. Previously, we demonstrated the efficacy and safety of tolvaptan in cirrhotic patients and showed that long‐term treatment improved survival.^11^, ^12^ Furthermore, rifaximin, a nonabsorbable antibiotic, was recommended for the treatment of hepatic encephalopathy and SBP in patients with cirrhosis. AKI has been reported to increase the mortality rate.^13^, ^14^ However, although the treatment options for cirrhosis have been improving, the efficacy of rifaximin treatment for AKI has not been elucidated. Therefore, it is necessary to reconsider the implications of AKI in patients with liver cirrhosis.

Here, we evaluated the effects of AKI on survival and considered the risk and protective factors of AKI in cirrhotic patients with ascites receiving tolvaptan therapy.

## Methods

### Patients and study design

This was a single‐center, retrospective observational study conducted between September 2013 and 2020. A total of 199 Japanese patients with liver cirrhosis (104 men, 52.3%), diagnosed based on imaging and biochemical results, complicated with ascites who received tolvaptan at 3.75–7.5 mg once per day (Samsca™; Otsuka Pharmaceutical Co. Ltd., Tokyo, Japan) were enrolled in the study. The patients were treated with conventional diuretics, including 0–160 mg/day furosemide and/or 0–400 mg/day spironolactone, and a salt‐restricted diet. Diagnosis of AKI and determination of the AKI grade were based on the KDIGO practice guidelines.^4^ Briefly, AKI was diagnosed based on an increase in serum creatinine concentration of ≥0.3 mg/dL from baseline within 48 h, an increase of 50% within 7 days, or urine volume < 0.5 mL/kg/h for 6 h.^4^ CKD was also diagnosed based on the KDIGO guidelines, with a disease stage higher than G3a (mild to moderate decrease in kidney function) and an estimated glomerular filtration rate (eGFR) < 60 mL/min/1.73 m^2^ defined as CKD.^15^ Survival rates and new onset of AKI were monitored for a median observation period of 8.1 (0.6–96.6) months.

This study was conducted in accordance with the principles of the Declaration of Helsinki and the ethics regulations of Tokyo Women's Medical University Hospital (TWMU, Tokyo, Japan). The TWMU Institutional Review Board approved the study protocol.

### Clinical parameters

The following baseline characteristics of the patients were assessed: age, sex, clinical history, underlying hepatic diseases, complications of cirrhosis (i.e., encephalopathy, esophageal/gastric varices, and hepatocellular carcinoma [HCC]), complications of diabetes mellitus, hypertension, CKD, SBP, cell‐free and concentrated ascites reinfusion therapy (CART) or ascites drainage, administration of diuretics, and treatment with ursodeoxycholic acid (UDCA), branched‐chain amino acids (BCAA), amino‐acid preparations for hepatic insufficiency, proton pump inhibitor (PPI)/H2 blockers, lactulose, kanamycin/rifaximin, carnitine, zinc agents, intestinal regulators, and/or laxatives. CART featured drainage of 3 L of ascites each time. Otherwise, the ascites drainage volume was 1–3 L each time, usually accompanied by intravenous albumin. Cases with suspected SBP did not undergo CART; however, all other cases were prescribed CART to minimize the use of blood products. Paracentesis was performed before and after tolvaptan treatment for patients whose ascites accumulations were severe. All patients received diuretics and medications for cirrhosis for more than 7 days. Blood samples for biochemistry and hematological data were collected at the time of administration of tolvaptan. Laboratory tests were performed to determine the serum concentrations of albumin, total bilirubin, aspartate aminotransferase, alanine transaminase, and γ‐glutamyl transferase; platelet count; fasting blood glucose; hemoglobin _A1c_; prothrombin time (PT%); PT international normalized ratio; blood urea nitrogen (BUN); creatinine; eGFR; cystatin C (Cys C); Cys C‐based glomerular filtration rate; uric acid; serum sodium and potassium; ammonia; C‐reactive protein; neutrophil‐to‐lymphocyte ratio; α‐fetoprotein (AFP); and des‐γ‐carboxy prothrombin (DCP). Child–Turcotte–Pugh (CTP)^16^ and model for end‐stage liver disease (MELD) scores^17^ were used for the evaluation of liver function.

### Follow‐up and outcomes

The patients were hospitalized and administered tolvaptan. All cases receiving tolvaptan were monitored 8 h later (or on the next day). After discharge, patients were followed up every 1–2 months at the outpatient clinic. Renal function was monitored at the clinic during regular follow‐up and blood samples were taken when patients visited the outpatient clinic because of illness. The prognosis was evaluated in terms of survival time until death or liver transplantation after tolvaptan treatment. The observation periods were from the date of initiation of tolvaptan to death, liver transplantation, and the time of censoring (January 2021).

### Statistical analysis

Data are presented as medians with minimum and maximum values. Significant differences between the AKI and non‐AKI groups were assessed with the Mann–Whitney U‐test or *χ* ^2^ test using SPSS statistical software (SPSS Inc., Chicago, IL, USA). Multivariate regression analysis was performed to compare the AKI and non‐AKI groups. Factors highly significant on univariate analysis were subjected to multivariate analysis. We assessed the variables by SPSS and there was no multicollinearity. The following factors were used to estimate odds ratios (ORs) and 95% confidence intervals (CIs): sex, hepatic encephalopathy, CKD, SBP, CART or ascites drainage, alubumin, alanine aminotransferase, hemoglobin _A1c_, BUN, creatinie, AFP, DCP, CTP score, and treatment with PPI/H2 blockers, and kanamycin/rifaximin. In all analyses, *P* < 0.05 was taken to indicate statistical significance. The survival rate and cumulative incidence of AKI were subjected to Kaplan–Meier analysis. Differences between groups were analyzed using a log‐rank test. Multivariate Cox regression analysis was used to evaluate the risk of AKI development based on the time to AKI occurrence after administration of tolvaptan and estimate hazard ratios (HRs) and 95% CIs.

## Results

### Baseline characteristics of patients treated with tolvaptan

The median age of the 199 patients receiving tolvaptan treatment was 61 (range 21–92) years, and 52.3% were male (Table 1). The underlying liver diseases included viral hepatitis (hepatitis C, 42 cases; hepatitis B, 12 cases), alcoholic liver disease/nonalcoholic fatty liver disease (62/25 cases), autoimmune hepatitis (2 cases), primary biliary cholangitis (19 cases), and primary sclerosing cholangitis (7 cases). With regard to complications of liver cirrhosis, hepatic encephalopathy (23.6%), esophageal/gastric varices (68.6%), and HCC (53.4%) were observed. All patients commenced on 3.75 mg/day tolvaptan; this was increased to 7.5 mg/day after 2–3 days in 139 (69.8%) patients who lacked adequate control of their body weight or urination. In six cases, tolvaptan was administered for more than 4 months and then stopped because the ascites had improved.

**Table 1:** Baseline characteristics of the patients and univariate and multivariate regression analyses of the risk of AKI

	Total (*n* = 199)	AKI (*n* = 46)	Non‐AKI (*n* = 153)	*P*‐value*	Odds ratio	95% CI	*P*‐value
Age (years)	61 (21–92)	60 (27–90)	62 (21–92)	0.73			
No. of males (%)	104 (52.3%)	19 (41.3%)	85 (55.6%)	0.09			
Underlying hepatitis				0.72			
Viral (HCV/HBV)	42/12	9/3	33/9				
Alcoholic/nonalcoholic	62/25	13/6	49/19				
AIH/PBC/PSC	2/19/7	0/7/2	2/12/5				
Others	35	8	27				
Complication type (%)							
Hepatic encephalopathy	47 (23.6%)	20 (43.5%)	27 (17.6%)	< 0.01	3.81	1.137–12.797	0.03
Esophageal/gastric varices	129 (68.6%)	29 (67.4%)	100 (69.0%)	0.85			
Diabetes mellitus	62 (31.2%)	12 (26.1%)	50 (32.7%)	0.40			
Hypertension	39 (19.6%)	11 (23.9%)	28 (18.3%)	0.40			
Hepatocellular carcinoma	52 (26.1%)	9 (19.6%)	43 (28.1%)	0.25			
Chronic kidney disease	102 (53.4%)	31 (70.5%)	71 (49.7%)	0.02			
Spontaneous bacterial peritonitis	37 (18.6%)	13 (28.3%)	24 (15.7%)	0.05			
Diuretics							
Furosemide dose (mg/day)	20 (0–160)	20 (0–120)	20 (0–160)	0.33			
Spironolactone dose (mg/day)	50 (0–400)	50 (0–150)	50 (0–400)	0.56			
Treatment (%)							
Ursodeoxycholic acid	120 (60.3%)	30 (65.2%)	90 (58.8%)	0.44			
Branched‐chain amino acids	136 (68.3%)	28 (60.9%)	108 (70.6%)	0.21			
Amino‐acid preparations for hepatic insufficiency	67 (33.7%)	17 (37%)	50 (32.7%)	0.59			
PPI/H2 blockers	148 (74.4%)	29 (63.0%)	119 (77.8%)	0.04	0.23	0.077–0.695	< 0.01
Lactulose	113 (56.8%)	27 (58.7%)	86 (56.2%)	0.77			
Kanamycin/rifaximin (Kanamycin *n* = 9, rifaximine *n* = 42^‡^)	48 (24.1%)	7 (15.2%)	41 (26.8%)	0.11	0.13	0.028–0.615	0.01
Carnitine	40 (20.1%)	13 (28.3%)	27 (17.6%)	0.12			
Zinc agents	22 (11.1%)	4 (8.7%)	18 (11.8%)	0.56			
Intestinal regulators	47 (23.6%)	11 (23.9%)	36 (23.5%)	0.96			
Laxative	62 (31.2%)	17 (37.0%)	45 (29.4%)	0.33			
Ascites treatment (%)							
CART or drainage	93 (46.7%)	30 (65.2%)	63 (41.2%)	< 0.01			
Laboratory data†							
Albumin (g/dL)	2.5 (1.5–4.4)	2.4 (1.5–3.3)	2.5 (1.5–4.4)	0.04			
Total bilirubin (mg/dL)	2.0 (0.3–27.3)	1.9 (0.3–27.3)	2.0 (0.3–24.2)	0.83			
Aspartate aminotransferase (U/L)	44 (10–551)	50 (14–164)	44 (10–551)	0.32			
Alanine aminotransferase (U/L)	26 (3–381)	25 (3–53)	26 (4–381)	0.03			
γ‐Glutamyl transpeptidase (U/L)	60 (9–926)	55 (9–359)	62 (10–926)	0.12			
Platelet counts (×10^4^/μL)	8.6 (1.5–42.4)	8.3 (2.1–39.8)	8.8 (1.5–42.2)	0.65			
Fasting blood glucose (mg/dL)	101 (58–364)	93 (64–364)	103 (58–305)	0.34			
Hemoglobin _A1c_ (%)	5.2 (3.4–11.6)	5.0 (3.8–6.6)	5.2 (3.4–11.6)	0.05			
Prothrombin time (PT; %)	56.1 (16.3–100.0)	56.4 (26.0–88.4)	56.0 (16.3–100.0)	0.49			
PT INR	1.31 (0.98–3.17)	1.28 (0.99–2.53)	1.32 (0.98–3.17)	0.41			
Blood urea nitrogen (mg/dL)	17.7 (3.8–74.9)	24.2 (5.7–61.8)	15.9 (3.8–74.9)	< 0.01			
Creatinine (mg/dL)	0.89 (0.35–3.30)	1.05 (0.40–3.16)	0.84 (0.35–3.30)	0.02			
eGFR (mL/min/1.73 m^2^)	60.0 (15.5–134.8)	46.7 (15.5–134.8)	62.5(16.8–133.6)	0.07			
Cystatin C (Cys C; mg/L)	1.3 (0.5–3.5)	1.56 (0.97–2.75)	1.26 (0.47–3.50)	0.17			
Cys C‐based GFR (mL/min/1.73 m^2^)	51.6 (13.5–171.8)	45.2 (18.7–75.8)	56.0 (13.5–171.8)	0.10			
Uric acid (mg/dL)	6.0 (1.4–14.2)	6.2 (3.4–14.2)	5.8 (1.4–12.6)	0.12			
Serum sodium (mEq/L)	137 (118–145)	138 (118–142)	137 (122–145)	0.94			
Serum potassium (mEq/L)	4.0 (1.3–6.1)	4.1 (1.4–5.5)	4.0 (1.3–6.1)	0.68			
Ammonia (μg/dL)	69 (15–499)	69 (24–269)	68 (15–499)	0.68			
C‐reactive protein (mg/dL)	1.09 (0.02–19.3)	1.73 (0.03–10.12)	0.93 (0.02–19.3)	0.18			
Neutrophil‐to‐lymphocyte ratio	3.42 (1.00–26.17)	4.42 (1.00–24.61)	3.18 (1.26–26.17)	0.63			
α‐Fetoprotein (ng/mL)	4 (1–4720)	3 (1–109)	4 (1–4720)	0.03			
Des‐γ‐carboxy prothrombin (mAU/mL)	58 (3–35 675)	35 (10–23 277)	66 (3–35 675)	0.95	1.00	1.000–1.000	0.02
Child–Turcotte–Pugh score	11 (6–15)	11 (7–15)	11 (6–15)	0.11			
MELD score	12 (2–35)	13 (2–35)	12 (3–29)	0.46			

* AKI *versus* non‐AKI.

^†^ At the initiation of tolvaptan.

^‡^ Kamaycin was switched to rifaximine in three patients.

AIH, autoimmune hepatitis; CART, cell‐free and concentrated ascites reinfusion therapy; CI, confidence interval; eGFR, estimated glomerular filtration rate; HBV, hepatitis B virus; HCC, hepatocellular carcinoma; HCV, hepatitis C virus; INR, international normalized ratio; MELD, model for end‐stage liver disease; *n*, number of patients; PBC, primary biliary cholangitis; PPI, proton pump inhibitor; PSC, primary sclerosing cholangitis.

### Characteristics of AKI cases and survival rates of cirrhosis according to type of complications

During the observation period, 46 cases (23.1%) suffered an AKI complication. The majority of these cases were classified as KDIGO stage 1 (38 cases, 82.6%). HRS‐AKI was observed in eight cases. The rates of hepatic encephalopathy (*P* < 0.01) and CKD (*P* = 0.02) were significantly higher in the AKI group compared to the non‐AKI group (Table 1). The rate of SBP was also higher in the AKI group (*P* = 0.05). In terms of the treatment of cirrhosis, the rate of PPI/H2 blocker treatment was significantly lower for AKI cases than for non‐AKI cases (*P* = 0.04). The rate of CART or ascites drainage was significantly higher for AKI cases than for non‐AKI cases (*P* < 0.01). With regard to biochemical findings, AKI cases exhibited lower levels of serum albumin (AKI vs. non‐AKI, 2.4 vs. 2.5 g/dL, respectively, *P* = 0.04), alanine aminotransferase (25 vs. 26 U/L, respectively, *P* = 0.03), and AFP (3 vs. 4 ng/mL, respectively, *P* = 0.03), and higher levels of BUN (24.2 vs. 15.9 mg/dL, respectively, *P* < 0.01) and creatinine (1.05 vs. 0.84 mg/dL, respectively, *P* = 0.02). Multivariate regression analysis indicated that hepatic encephalopathy (OR 3.81, 95% CI 1.137–12.797, *P* = 0.03) was positively associated with the occurrence of AKI, whereas treatment with PPI/H2 blocker (OR 0.23, 95% CI 0.077–0.695, *P* < 0.01) or kanamycin/rifaximin (kanamaycin 9 cases and rifaximin 42 cases. Kanamycin was switched to rifaximin in three cases. OR 0.13, 95% CI 0.028–0.615, *P* = 0.01) were negatively associated.

**Figure 1:**
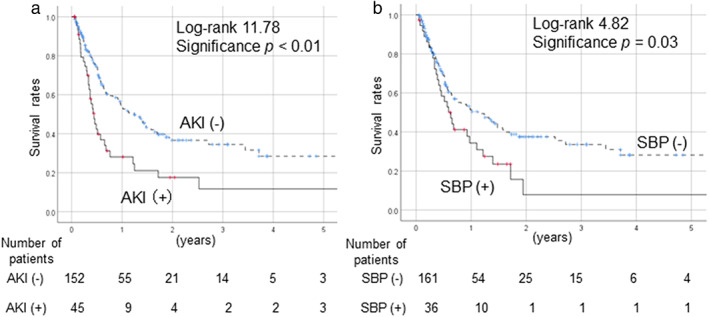
Mortality rates of cirrhotic patients with and without complications. (a) AKI. (b) SBP. (a) Patients with AKI had a significantly poorer prognosis than patients without AKI (*P* < 0.01). (b) Patients with SBP had significantly poorer survival compared to non‐SBP cases (*P* = 0.03). AKI, acute kidney injury; SBP, spontaneous bacterial peritonitis.

The survival rates of cirrhotic patients after tolvaptan administration, as examined via a Kaplan–Meier analysis, are shown in Figure 1. Prognosis was significantly poorer in patients with AKI than in patients without AKI (*P* < 0.01) (Fig. 1a) and in SBP cases than in the non‐SBP cases (*P* = 0.03) (Fig. 1b). After the exclusion of HCC cases, AKI and SBP were also associated with significantly poorer outcomes in comparison to non‐AKI and non‐SBP cases, respectively.

### Incidence of AKI according to the type of complications and treatment

**Figure 2:**
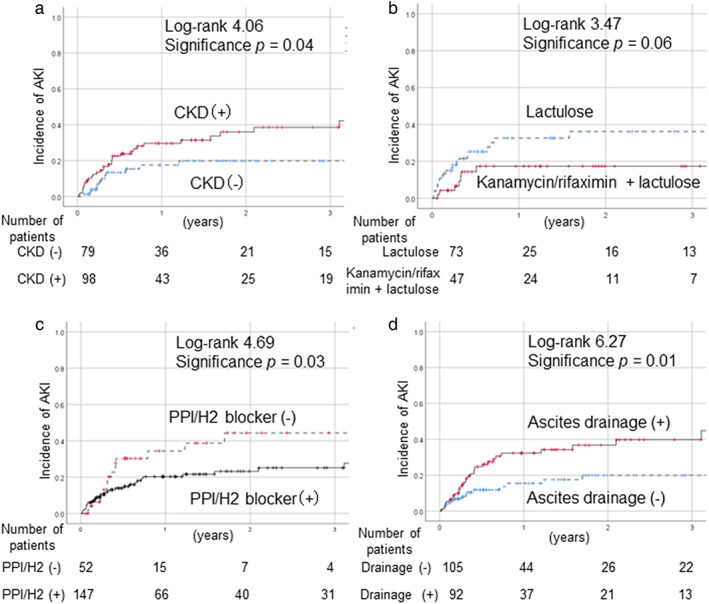
Incidence of AKI according to complication type or treatment. (a) CKD. (b) Treatment with lactulose versus kanamycin/rifaximin + lactulose. (c) Treatment with PPI/H2 blockers. (d) Results of ascites drainage as evaluated using Kaplan–Meier analysis. (a) The incidence of AKI was significantly higher in patients with CKD compared to the non‐CKD cases (*P* = 0.04). (b) In the comparison of treatment with lactulose or kanamycin/rifaximin with lactulose, the rate of AKI was lower in the kanamycin/rifaximin treatment group among patients with hepatic encephalopathy (*n* = 122, *P* = 0.06). (c) Treatment with PPI/H2 blockers significantly decreased the rate of AKI (*P* = 0.03). (d) CART or ascites drainage was significantly associated with an increased rate of AKI (*P* = 0.01). AKI, acute kidney injury; CART, cell‐free and concentrated ascites reinfusion therapy; CKD, chronic kidney disease; PPI, proton pump inhibitor.

Although diabetes mellitus as a complication was not associated with an increased rate of developing AKI, SBP was more frequent in the AKI group (*P* = 0.05). The incidence of AKI was significantly higher in patients with CKD compared to non‐CKD cases (*P* = 0.04) (Fig. 2a). We compared the AKI cases with and without CKD on Table S1, Supporting information. CKD cases were significantly older; however, no other factor (excluding renal factors) differed significantly between the groups. With regard to the treatment of cirrhosis, there were no significant differences in rates of treatment with UDCA, BCAA, or other agents for lowering ammonia between the AKI group and the non‐AKI group. Among patients with hepatic encephalopathy (*n* = 122), the incidence of AKI tended to be lower in patients treated with kanamycin/rifaximin with lactulose compared to those treated with lactulose alone (*P* = 0.06) (Fig. 2b). Treatment with PPI/H2 blockers significantly decreased the rate of AKI onset (*P* = 0.03) (Fig. 2c), whereas the onset rate was significantly elevated in patients who underwent CART or ascites drainage (*P* = 0.01) (Fig. 2d).

### Risk factors for AKI development in patients with decompensated cirrhosis

We evaluated the factors that were predictive of AKI development in patients with cirrhosis via multivariate analysis using a Cox hazard model. The parameters included were sex, hepatic encephalopathy, CKD, SBP, CART or ascites drainage, alubumin, alanine aminotransferase, hemoglobin _A1c_, BUN, creatinie, AFP, DCP, CTP score, and treatment with PPI/H2 blockers, and kanamycin/rifaximin (Table 2). The incidence of AKI was significantly higher in patients with hepatic encephalopathy (HR 4.18, 95% CI 1.618–10.771, *P* < 0.01). By contrast, the incidence of AKI was significantly lower in patients with a higher serum albumin level (HR 0.36, 95% CI 0.142–0.914, *P* = 0.03). Treatment with PPI/H2 blockers (HR 0.30, 95% CI 0.126–0.711, *P* < 0.01) or kanamycin/rifaximin (HR 0.26, 95% CI 0.075–0.929, *P* = 0.04) was significantly associated with a reduced risk of AKI development.

**Table 2:** Predictors of AKI in cirrhotic patients based on Cox multivariate analysis

Variable	Hazard ratio	95% CI	*P*‐value
Hepatic encephalopathy	4.18	1.618–10.771	<0.01
Albumin (g/dL)	0.36	0.142–0.914	0.03
Des‐γ‐carboxy prothrombin (DCP; mAU/mL)	1.00	1.000–1.000	0.02
PPI/H2 blocker treatment	0.30	0.126–0.711	<0.01
Kanamycin/rifaximin treatment	0.26	0.075–0.929	0.04

Factors analyzed: sex, hepatic encephalopathy, chronic kidney disease, spontaneous bacterial peritonitis, CART or ascites drainage, alubumin, alanine aminotransferase, hemoglobin _A1c_, blood urea nitrogen, creatinie, α‐fetoprotein, DCP, CTP score, and treatment with PPI/H2 blockers, and kanamycin/rifaximin.

AKI, acute kidney injury; CI, confidence interval, PPI, proton pump inhibitor.

## Discussion

The results of this study indicate that the onset of AKI is associated with a poorer outcome in liver cirrhosis patients with ascites and the incidence of AKI is increased in patients with encephalopathy and decreased liver function. By contrast, PPI/H2 blocker or kanamycin/rifaximin treatment appeared to decrease the risk of AKI in these patients.

Our study population consisted of patients with decompensated cirrhosis requiring tolvaptan treatment. We previously reported the safety and efficacy profiles of long‐term tolvaptan.^12^, ^18^ Most patients had to remain on medication because of liver cirrhosis progression. Only six patients could cease treatment. In the tolvaptan era, it is essential to monitor the clinical courses of patients with liver cirrhosis who are on tolvaptan and to seek always to improve treatment. Hiramine et al.^19^ reported that the AKI incidence was significantly lower in patients treated with tolvaptan than in those not so treated (27.6% vs. 44.7%, respectively, *P* = 0.028). While renal function worsened with higher natriuretic agent doses in the control group, Mori et al.^20^ reported that renal blood flow was maintained in patients on tolvaptan. In addition, neurohormonal factors and blood pressure were not markedly altered, with this outcome thought to protect against worsening renal function. In the present study, 46 (23.1%) patients developed AKI after treatment with tolvaptan. Although there were no significant differences in the doses of other diuretics between the AKI and non‐AKI groups, the rates of hepatic encephalopathy and CKD were significantly higher in patients with AKI in the present study. The recent guidelines suggest that large amounts of diuretics should not be used to avoid renal dysfunction.^10^ Therefore, the rates of treatment with other fundamental diuretics were extremely low and were thought not to be associated with the development of AKI in our study population. Patients with AKI had significantly poorer prognosis even after the exclusion of cases of HCC from our cohort, consistent with the results of previous studies.^19^, ^21^, ^22^

The creatinine level and the eGFR are commonly used to evaluate kidney function. Cys C enhanced the renal function in cirrhosis patients.^23^ We found that the cys C level and cys C‐based GFR was not associated with AKI complications. Type 1 HRS, the prototype of acute renal dysfunction in cirrhosis, has been re‐named AKI‐HRS,^5^ and was observed in eight cases.

Ascites were the most common complication of cirrhosis (86.5%), followed by hepatic encephalopathy (37.8%), variceal bleeding (17.5%), HRS (6.3%), and SBP (6.1%).^24^ Rifaximin was introduced to treat hepatic encephalopathy by reducing ammonia via regulation of the gut microbiome. Dong et al.^25^ reported that long‐term rifaximin treatment reduced the rate of AKI in cirrhosis patients. However, reports on this topic are limited. In the present study, kanamycin/rifaximin treatment with lactulose tended to decrease the incidence of AKI compared to treatment with lactulose alone in cases with hepatic encephalopathy (*P* = 0.06). A leaky gut increases the risk of SBP in patients with ascites, and kanamycin/rifaximin has been reported to reduce SBP in such cases^26^ In patients receiving treatment for hepatic encephalopathy, Zeng et al.^27^ reported that kanamycin/rifaximin protected against ascites exacerbation (*P* < 0.001) and gastric variceal bleeding (*P* = 0.03).

PPI treatment has been reported to have negative effects in patients with cirrhosis.^28^, ^29^ Patients receiving PPIs and/or SBP prophylaxis were found to have an increased risk of infection.^28^ In a systematic review and meta‐analysis, the pooled ORs for hepatic encephalopathy and SBP were 2.31 (95% CI 1.63–3.28) and 1.72 (95% CI 1.42–2.09), respectively, and these were significantly higher in PPI users compared with non‐PPI users.^29^ However, PPI treatment was reported to reduce the risk of post‐endoscopic variceal band ligation bleeding.^30^ In the present study, the rate of treatment with PPI/H2 blockers was significantly lower in AKI cases (63.0%) compared to non‐AKI cases (77.8%). The HR associated with developing AKI was 0.30 (95% CI 0.126–0.711, *P* < 0.01). We speculated that PPI treatment can reduce variceal bleeding, thus decreasing the risk of AKI by exerting protective effects on renal blood flow. Further studies are required to determine the mechanism underlying the protective effect of PPIs against AKI. Moreover, the combination of PPI and kanamycin/rifaximin treatment may help to decrease the incidence of AKI.

**Figure 3:**
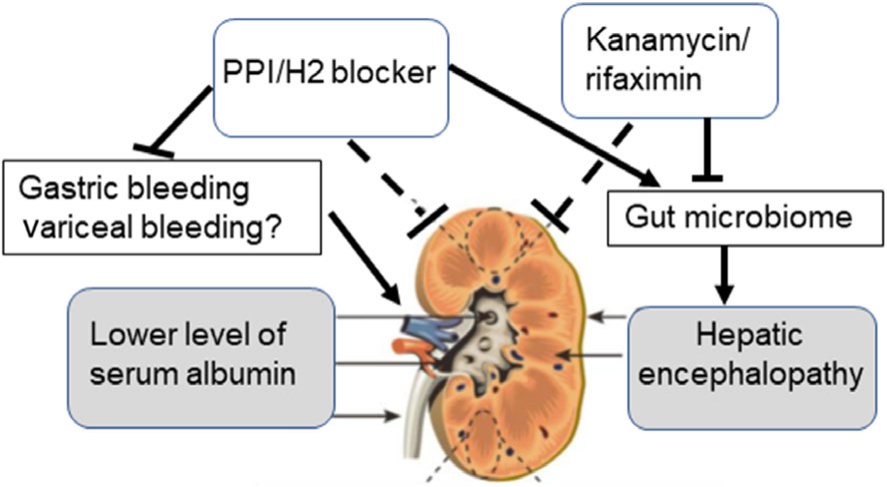
Development of AKI in cirrhosis. In patients who developed decompensated cirrhosis with ascites, encephalopathy, and poor liver function were associated with a higher incidence of AKI, whereas treatment with PPI/H2 blockers or kanamycin/rifaximin reduced the incidence of AKI. AKI, acute kidney injury; PPI, proton pump inhibitor.

Ascites drainage was performed in cases with severe ascites accumulation. Post‐ascites drainage, AKI was reported in 10.9% of the cases, despite adequate colloid replacement.^31^ In the present study, CART or ascites drainage was performed in 65.2% of the patients in the AKI group and 41.2% of the patients in the non‐AKI group (*P* < 0.01). CART or ascites drainage was associated with the development of AKI and should therefore be avoided. However, this did not remain a risk factor for AKI after multivariate analysis. Hence, we hypothesize that AKI development is associated with cirrhosis (Fig. 3). In patients who developed decompensated cirrhosis with ascites, encephalopathy and poor liver function were associated with a higher incidence of AKI, whereas treatment with PPI/H2 blockers and kanamycin/rifaximin altered the gut microbiome and might have reduced the incidence of AKI.

The present study had some limitations. The study was retrospective, and we did not perform gut microbiome analysis. There might have been physician‐based or institution‐based bias in the treatment of cirrhotic patients. AKI was evaluated specifically in cirrhotic patients undergoing treatment with tolvaptan. This limited the generalizability of our results; however, the disease conditions (e.g., decompensation level) were similar across the included patients, and thus, fair comparisons between patient groups could be made. The effects of drug combinations on cirrhosis should be evaluated in future prospective studies.

In conclusion, AKI was associated with an increased mortality rate in patients with liver cirrhosis. In patients who develop decompensated liver cirrhosis with ascites, treatment with PPI/H2 blockers and kanamycin/rifaximin may reduce the incidence of AKI.

## Human/animal rights

All procedures were performed in accordance with the ethical standards of the 1964 Declaration of Helsinki and its later amendments.

## Informed consent

Informed consent was obtained from the patients.

## Supporting information


**Table S1.** AKI patients with and without CKD.

## Data Availability

The datasets used and/or analyzed in the current study are available from the corresponding author upon reasonable request.
