# Supplementary material for: Risk and protective factors of acute kidney injury in decompensated cirrhotic patients with ascites on tolvaptan
Source: JGH Open. 2021 Nov 1;5(11):1298–305. doi: 10.1002/jgh3.12672 (PMC8593781; doi:10.1002/jgh3.12672)
Supplement: Supplementary file 1 — Table S1. AKI patients with and without CKD. [file JGH3-5-1298-s001.docx]

Spplemental Table 1. AKI patients with and without CKD

|  | AKI without CKD  (*n* = 15) | AKI with CKD  (*n* = 31) | *p*-value |
| --- | --- | --- | --- |
| Age (years) | 54 (27–70) | 61 (29–90) | 0.04 |
| No. of males (%) | 7 (46.7%) | 12 (38.7%) | 0.61 |
| Underlying hepatitis |  |  | 0.56 |
| Viral (HCV/HBV) | 3/2 | 6/1 |  |
| Alcoholic/nonalcoholic | 5/2 | 8/4 |  |
| AIH/PBC/PSC | 0/1/2 | 0/6/0 |  |
| Others | 1 | 7 |  |
| Complication type (%) |  |  |  |
| Hepatic encephalopathy | 6 (40.0%) | 14 (45.2%) | 0.74 |
| Esophageal/gastric varices | 11 (73.3%) | 18 (58.1%) | 0.31 |
| Diabetes mellitus | 3 (20.0%) | 9 (29.0%) | 0.51 |
| Hypertension | 3 (20.0%) | 8 (25.8%) | 0.67 |
| Hepatocellular carcinoma | 4 (26.7%) | 5 (16.1%) | 0.40 |
| Spontaneous bacterial peritonitis | 5 (33.3%) | 8 (25.8%) | 0.60 |
| Diuretics |  |  |  |
| Furosemide dose (mg/day) | 20 (0–40) | 20 (0–120) | 0.16 |
| Spironolactone dose (mg/day) | 50 (0–150) | 50 (0–150) | 0.59 |
| Treatment (%) |  |  |  |
| Ursodeoxycholic acid | 10 (66.7%) | 20 (64.5%) | 0.89 |
| Branched-chain amino acids | 10 (66.7%) | 18 (58.1%) | 0.58 |
| Amino-acid preparations for hepatic insufficiency | 5 (33.3%) | 12 (38.7%) | 0.72 |
| PPI/H2 blockers | 11 (73.3%) | 18 (58.1%) | 0.31 |
| Lactulose | 9 (60.0%) | 18 (58.1%) | 0.90 |
| Kanamycin/rifaximin | 3 (20.0%) | 4 (12.9%) | 0.53 |
| Carnitine | 4 (26.7%) | 9 (29.0%) | 0.92 |
| Zinc agents | 1 (6.7%) | 3 (9.7%) | 0.73 |
| Intestinal regulators | 2 (13.3%) | 9 (29.0%) | 0.24 |
| Laxative | 5 (33.3%) | 12 (38.7%) | 0.72 |
| Ascites treatment (%) |  |  |  |
| CART or drainage | 10 (66.7%) | 20 (64.5%) | 0.89 |
| **Laboratory data** |  |  |  |
| Albumin (g/dL) | 2.5 (1.5–2.9) | 2.1 (1.6–3.3) | 0.39 |
| Total bilirubin (mg/dL) | 2.9 (0.6–27.3) | 1.8 (0.3–13.9) | 0.24 |
| Aspartate aminotransferase (U/L) | 50 (27–110) | 49 (14–164) | 0.36 |
| Alanine aminotransferase (U/L) | 29 (13–51) | 23 (3–53) | 0.38 |
| γ-Glutamyl transpeptidase (U/L) | 63 (17–336) | 48 (9–359) | 0.12 |
| Platelet counts (×10^4^/μL) | 9.7 (3.2–15.5) | 7.9 (2.1–39.8) | 0.64 |
| Fasting blood glucose (mg/dL) | 91 (64–111) | 95 (67–364) | 0.14 |
| Hemoglobin _A1c_ (%) | 4.7 (3.8–6.1) | 5.1 (3.9–6.6) | 0.28 |
| Prothrombin time (PT; %) | 64.8 (26.0–88.4) | 56.0 (27.8–81.4) | 0.54 |
| PT INR | 1.20 (1.02–2.16) | 1.30 (0.99–2.53) | 0.60 |
| Blood urea nitrogen (mg/dL) | 15.0 (5.7–34.1) | 32.5 (11.7–61.8) | <0.01 |
| Creatinine (mg/dL) | 0.72 (0.40–0.91) | 1.29 (0.93–3.16) | <0.01 |
| eGFR (mL/min/1.73 m^2^) | 79.3 (60.1–134.8) | 38.4 (15.5–55.6) | <0.01 |
| Cystatin C (Cys C; mg/L) | 1.01 (0.97–1.03) | 1.73 (1.23–2.75) | <0.01 |
| Cys C-based GFR (mL/min/1.73 m^2^) | 72.5 (65.1–75.8) | 36.2 (18.7–60.4) | <0.01 |
| Uric acid (mg/dL) | 5.1 (4.8–8.8) | 6.6 (3.4–14.2) | 0.21 |
| Serum sodium (mEq/L) | 139 (135–142) | 137 (118–142) | 0.01 |
| Serum potassium (mEq/L) | 3.9 (2.9–4.5) | 4.3 (1.4–5.5) | 0.07 |
| Ammonia (μg/dL) | 66 (29–116) | 72 (24–269) | 0.33 |
| C-reactive protein (mg/dL) | 1.89 (0.25–7.02) | 1.73 (0.03–10.12) | 0.85 |
| Neutrophil-to-lymphocyte ratio | 4.34 (1.57–8.50) | 4.57 (1.00–24.61) | 0.22 |
| α-Fetoprotein (ng/mL) | 3 (2–109) | 3 (1–37) | 0.36 |
| Des-γ-carboxy prothrombin (mAU/mL) | 34 (11–1,673) | 59 (10–23,277) | 0.29 |
| Child–Turcotte–Pugh score | 11 (7–14) | 11 (9–15) | 0.02 |
| MELD score | 9 (2–20) | 14 (7–35) | 0.61 |

AIH, autoimmune hepatitis; CART, cell-free and concentrated ascites reinfusion therapy; CI, confidence interval; eGFR, estimated glomerular filtration rate; HBV, hepatitis B virus; HCC, hepatocellular carcinoma; HCV, hepatitis C virus; INR, international normalized ratio; *n*, number of patients; MELD, model for end-stage liver disease; PBC, primary biliary cholangitis; PPI, proton pump inhibitor; PSC, primary sclerosing cholangitis.
